# Opioid Overdose Hospitalizations During COVID-19: The Experience of Pennsylvania

**DOI:** 10.1177/11782218231222343

**Published:** 2024-01-10

**Authors:** Chan Shen, James Douglas Thornton, Ning Li, Shouhao Zhou, Li Wang, Douglas L. Leslie, Sarah S. Kawasaki

**Affiliations:** 1 Department of Surgery, The Pennsylvania State University, College of Medicine, Hershey, PA, USA; 2 Department of Public Health Sciences, The Pennsylvania State University, College of Medicine, Hershey, PA, USA; 3 Department of Pharmaceutical Health Outcomes and Policy, College of Pharmacy, University of Houston, TX, USA; 4 Department of Economics and Finance, Salisbury University, Salisbury, MD, USA; 5 Penn State Health Milton S. Hershey Medical Center, Hershey, PA, USA

**Keywords:** Opioid overdose, hospitalization, COVID-19

## Abstract

**Objective:**

The COVID-19 pandemic placed extreme burden on hospitals, while opioid overdose is another challenging public health issue. This study aimed to examine the trends and outcomes of opioid overdose hospitalizations in Pennsylvania during 2018 to 2021.

**Design:**

We identified opioid overdose hospitalizations in the state of Pennsylvania using the state-wide hospital discharge database (PHC4) 2018 to 2021. We examined the number of opioid overdose hospitalizations, the corresponding mortality and discharges against medical advice comparing the pre-COVID period (2018-2019) and the COVID period (2020-2021). We also assessed what patient and hospital characteristics were associated with in-hospital death or leaving against medical advice.

**Results:**

A total of 13 446 opioid-related hospitalizations were identified in 2018 to 2021. Compared to pre-pandemic, a higher percentage of cases involving synthetics (17.0%vs 10.3%, *P* < .0001) were observed during COVID. After controlling for covariates, there was no significant difference in opioid overdose in-hospital deaths in the years 2020 to 2021 compared to 2018 to 2019 (OR = 0.846, 95% CI: 0.71-1.01, *P* = .065). The COVID period was significantly associated with more leaving against medical advice compared to years 2018 to 2019 (OR = 1.265, 95% CI: 1.11-1.44, *P* = .0003). Compared to commercial insurance, Medicaid insurance was associated with higher odds of both in-hospital death (OR = 1.383, 95% CI: 1.06-1.81, *P* = .0176) and leaving against medical advice (OR = 1.903, 95% CI: 1.56-2.33, *P* < .0001).

**Conclusion:**

There were no substantial changes in the number of overall opioid overdose cases and deaths at hospitals following the outbreak of COVID-19 in Pennsylvania. This observation suggests that an increased number of patients may have succumbed to overdoses outside of hospital settings, possibly due to a higher severity of overdoses. Further, we found that patients were more likely to leave against medical advice during the COVID-19 pandemic.

## Introduction

The prevalence of drug overdoses and resultant fatalities have notably escalated since the onset of the COVID-19 pandemic. Several region-centric studies have recorded an upward trend in opioid overdoses that began at the early onset of COVID-19.^1-3^ The Centers for Disease Control and Prevention (CDC) has been collecting mortality data related to COVID-19 and has also identified a similar surge in opioid overdose deaths nationwide. Provisional statistics by the CDC suggested a 31% increase in opioid overdose mortality rate in 2020 compared to 2019,^4^ and an estimated more than 107 000 deaths were attributed to opioid overdose in the 12-month period ending August 2022.^5^ The disruptions owing to the COVID-19 pandemic imposed many socioeconomic stressors, including social and physical isolation, economic insecurity and restricted access to treatment of substance use disorders,^1,6,7^ which worsen mental health and substance use,^1,8,9^ especially when fentanyl and other synthetic opioids gradually started contaminating the illicit drug supply.^10^ Moreover, drastic changes in social support structures during COVID-19, for example reduced harm reduction services, may have further exacerbated the drug overdose epidemic.^2,11^

While some studies reported a surge in emergency department visits for drug overdose during COVID,^2,12^ the trend of opioid overdose incidences and outcomes at hospital inpatient settings have not been well studied. The COVID-19 pandemic placed immense pressure on hospitals.^13-15^ A study of 625 US hospitals found that more than 60% of these hospitals experienced ED or ICU overcrowding and approximately half reported ventilator shortages during COVID.^14^ A survey of frontline healthcare providers from 77 countries and other similar studies revealed that shortages of ICU nurses, intensivists, ICU beds, and personal protective equipment prevailed worldwide including in the United States.^13,16,17^ Moreover, several studies involving US health care professionals reported a remarkably high prevalence of emotional burnout in this group, with rates as high as 58%.^13,18,19^ With hospitals overwhelmed with COVID, the impact on treatment outcomes of opioid overdose cases presenting in these same hospitals is unknown. Further, how patient and hospital characteristics are associated with opioid overdose outcomes is understudied. Leaving against medical advice was more likely to occur during the peak of COVID-19^20^ and it put patients at great risk of readmission and mortality.^21,22^ Factors that contributed to higher likelihood of leaving against medical advice during the pandemic have not been examined, and might be related to an increase in the overdose deaths.

This study aimed to examine the trends and outcomes of opioid overdose hospitalizations in Pennsylvania during 2018 to 2021. The primary outcomes assessed were cases of opioid overdose, related fatalities in hospitals, and discharges against medical advice. We also investigated how the trends and outcomes were associated with patient and hospital characteristics.

## Methods

### Data source and study cohort

The data source for this study was the Pennsylvania Health Care Cost Containment Council (PHC4) hospital discharge database from year 2018 to 2021. PHC4 is a state agency that collects data on inpatient admissions to all hospitals and surgical facilities in Pennsylvania on a quarterly basis. The database includes patient demographics, diagnosis codes, procedure codes, and hospital information. In this study, we selected admissions for adult patients aged 18 and above with primary International Classification of Diseases (ICD-10) diagnosis codes indicating opioid overdose. We used a widely accepted list of codes to identify opioid overdose including: T400 (Opium), T401 (Heroin), T402 (Natural/semi-synthetic opioids), T403 (Methadone), T404 (Synthetic narcotics), T405 (Cocaine), and T406 (Other Unspecified Narcotics).^23-25^ The study was exempted by the institutional review board because it used de-identified secondary data.

### Outcomes

The primary outcome of interest was opioid overdose and related in-hospital death. Death was ascertained based on the discharge status showing “expired.” A secondary outcome was leaving against medical advice also based on discharge status. The PHC4 data only specifies the quarter of admission, not the exact date due to confidentiality issues. Therefore, we used quarters and years to examine the temporal trends of opioid overdose during our study period.

### Covariates

One key variable that we examined was the year of admission, which was stratified into pre-COVID-19 period (years 2018-2019) and COVID-19 period (years 2020-2021). We considered patient demographics including patient age, sex, race/ethnicity, and rural-urban status. Age was categorized into the following groups: ⩽44 years, 45-64 years, ⩾65 years. Race/ethnicity was categorized as Hispanic, Non-Hispanic White, Non-Hispanic Black, and Other Non-Hispanic. Rural-urban status based on the county of residence of the subjects was determined by Rural-Urban Continuum Code (RUCC), which was categorized into: Counties with Metro areas of 1 million population or more, Counties in Metro areas of 250 000 to 1 million population, Counties in Metro areas of fewer than 250 000 population, and Non-Metro Areas. We also considered hospital size based on the number of hospital beds, which was categorized into 4 groups: 1000 or more beds, 500 to 999 beds, 100 to 500 beds, and 100 or fewer beds. Distance to the hospital was calculated based on the geographical distance between patient residence zip code and hospital zip code. Opioid use type was summarized into 4 categories based on the primary diagnosis code (PDX in the database): natural/semi-synthetic opioids (T402), heroin (T401), synthetics (T204), others (T400, T403, T406). We also examined co-existing alcohol use (T510, T519) and stimulant use (T436).

### Statistical analysis

We provided descriptive statistics including frequencies and percentages for categorical variables and the means and standard deviations (SDs) for continuous variables. Statistical differences by pre-COVID versus during COVID periods were examined using the χ^2^ test for categorical variables and Student *t*-test for continuous variables. Two separate multivariable logistic regressions were conducted to evaluate the association between the covariates and 2 binary outcomes: opioids overdose death and leaving against medical advice. Notice that the logistic regression for leaving against medical advice was performed only on cases that did not lead to death, law enforcement, and hospice as these are unlikely to be patient choices. The adjusted odds ratios (OR), and 95% confidence intervals (95% CI) and p-values were provided. A *P*-value of <.05 was considered statistically significant. All statistical analyses were performed using the SAS statistical package (version 9.4; SAS Institute, Inc., Cary, NC, USA).

## Results

Table 1 provides the patient characteristics of our study sample stratified by the year of hospitalization: 2018 to 2019versus 2020 to 2021. The study included 13 446 opioid-related hospitalizations in total. The majority of cases involved males and the proportion of male patients increased significantly from 60.3% in 2018 to 2019 to 63.9% in 2020 to 2021 (*P* < .0001). Non-Hispanic White patients were the majority although the proportion decreased from 62.9% in 2018 to 2019 to 58.1% in 2020 to 2021, which is statistically significant at *P* < .0001. Medicaid and Medicare together accounted for the largest proportion of admissions. We observed a significantly higher proportion of Medicaid admissions in the 2020 to 2021 group than in the 2018 to 2019 group and a lower proportion of Medicare admissions (56.7%vs 52.8% for Medicaid and 24.5%vs 27.5% for Medicare). Hospitals with 100 to 500 beds were the most common size in both periods, although there was a significantly higher proportion in the 2020 to 2021 group (65%vs 55.9%, *P* < .0001). The majority of admissions were from metropolitan areas with a population of 1 million or more (68.4% and 66.1% in 2018-2019 and 2020-2021). We did not observe any significant change in the distance from patient residence to hospital (*P* = .471). The proportion of emergency visits increased significantly from 90.8% in 2018 to 2019 to 94.0% in 2020 to 2021 (*P* < .0001).

**Table 1.** Patients characteristics for opioid overdose hospitalizations.

	Year		*P-*value
	2018-2019 (N = 6963)	2020-2021 (N = 6483)	
Age categories, n (%)			.3519^a^
45-64 y	3041 (44.3%)	2776 (43.5%)	
⩽44 y	2913 (42.5%)	2718 (42.6%)	
⩾65 y	905 (13.2%)	893 (14.0%)	
Gender, n (%)			<.0001^a^
Male	4202 (60.3%)	4142 (63.9%)	
Female	2761 (39.7%)	2341 (36.1%)	
Race/Ethnicity, n (%)			<.0001^a^
Hispanic	378 (5.4%)	482 (7.4%)	
White Non-Hispanic	4380 (62.9%)	3765 (58.1%)	
Black Non-Hispanic	1924 (27.6%)	1854 (28.6%)	
Other Non-Hispanic	281 (4.0%)	381 (5.9%)	
Insurance type—first digit, n (%)			<.0001^a^
Commercial	959 (13.8%)	862 (13.3%)	
Government	61 (0.9%)	66 (1.0%)	
Medicaid	3677 (52.8%)	3675 (56.7%)	
Medicare	1913 (27.5%)	1587 (24.5%)	
Uninsured	258 (3.7%)	255 (3.9%)	
Unknown/Missing	95 (1.4%)	38 (0.6%)	
Number of hospital beds, n (%)			<.0001^a^
100-500 beds	4527 (65.0%)	3626 (55.9%)	
100 or less beds	258 (3.7%)	235 (3.6%)	
1000 or more bed	352 (5.1%)	240 (3.7%)	
500-999 beds	1826 (26.2%)	2382 (36.7%)	
Rural-Urban Continuum Code 2013, n (%)			.0091^a^
Counties in metro areas of 1 million population or more	4764 (68.4%)	4285 (66.1%)	
Counties in metro areas of 250 000 to 1 million population	1538 (22.1%)	1486 (22.9%)	
Counties in metro areas of fewer than 250 000 population	424 (6.1%)	470 (7.2%)	
Non-metro areas	237 (3.4%)	242 (3.7%)	
Distance to hospital from home			.4708^b^
Mean (SD)	7.4 (13.94)	7.2 (13.20)	
Median (Range)	3.7 (0.0, 298.5)	3.5 (0.0, 245.7)	
IQR	1.6, 7.6	1.7, 7.6	
Types of opioids, n (%)			<.0001^a^
Natural/semi-synthetic opioids	1673 (34.7%)	1793 (38.3%)	
Heroin	1921 (39.8%)	1491 (31.9%)	
Synthetics	495 (10.3%)	793 (17.0%)	
Others	737 (15.3%)	599 (12.8%)	
Alcohol, n (%)			.7603^a^
No	6816 (97.9%)	6351 (98.0%)	
Yes	147 (2.1%)	132 (2.0%)	
Stimulant, n (%)			.0535^a^
No	6761 (97.1%)	6257 (96.5%)	
Yes	202 (2.9%)	226 (3.5%)	
Discharge status, n (%)			<.0001^a^
Dead	385 (5.5%)	371 (5.7%)	
Hospice	58 (0.8%)	60 (0.9%)	
Law enforcement	70 (1.0%)	56 (0.9%)	
Left against medical advice or discontinued care	821 (11.8%)	900 (13.9%)	
Transferred to other hospitals	369 (5.3%)	345 (5.3%)	
Others/Not determine elsewhere	686 (9.9%)	735 (11.3%)	
Psychiatric hospital	533 (7.7%)	427 (6.6%)	
Routine discharge/self care	3704 (53.2%)	3335 (51.4%)	
Skilled nursing facilities	337 (4.8%)	254 (3.9%)	

a Chi-Square *P*-value.

b Unequal variance 2 sample *t*-test.

**Figure 1.**
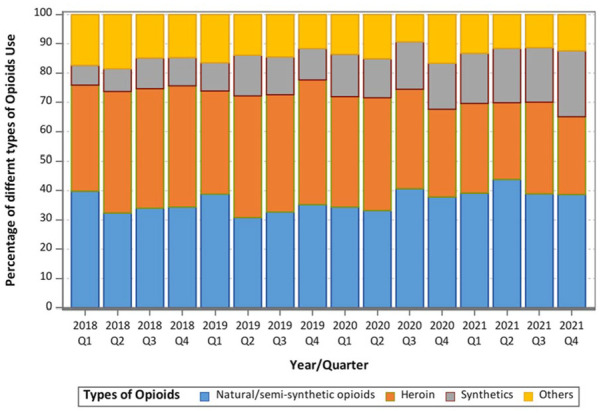
Different types of opioids overdose use (100% Stacked) across quarter for the year 2018 to 2021. Stacked bar graph showing how patients are distributed among different types of opioid use across years, and there is an increase trend of synthetics use overtime. Less than half of patients used the Natural/Semi-Synthetic Opioids. Blue denotes Natural/Semi-Synthetic Opioids, orange denotes patients who used Heroin, gray denotes patients who used Synthetics Opioids, and yellow denotes Other Opioids.

An alarmingly higher percentage of cases involved synthetics in 2020 to 2021 compared to 2018 to 2019 (17.0%vs 10.3%, *P* < .0001). To further examine the trends, we created a stacked bar chart (Figure 1) showing the types of opioids used over time by quarters from the beginning of 2018 to the end of 2021. The chart illustrates the substantive increase in synthetics with a simultaneous decrease in heroin and other opioids use, while overdose due to natural or semi-synthetic opioids were relatively stable.

The results from the multivariable logistic regression for overdose death are presented in Table 2. Younger age (⩽44 years) was significantly associated with higher odds of death (OR = 1.469, 95% CI: 1.00-2.15, *P* = .0484) compared to older age (⩾65 years). Males were less likely to die (OR = 0.827, 95% CI: 0.69-0.99, *P* = .0416). Patients with Medicaid insurance were more likely to die from opioids overdose compared to patients with commercial insurance (OR = 1.383, 95% CI: 1.06-1.81, *P* = .0176). Overdose of synthetic opioids were significantly associated with higher odds of death compared to other types of opioids use (OR = 1.422, 95% CI: 1.10-1.85, *P* = .0083). Co-existing alcohol use was associated with lower odds of overdose death (OR = 0.359, 95% CI: 0.13-0.98, *P* = .0453). There was no significant difference in opioids overdose hospitalization death in the years 2020 to 2021 compared to 2018 to 2019 after controlling for all the other factors.

**Table 2.** Logistic Regression for Death among Opioid Overdose Hospitalizations.

Parameter		Odds ratios	95% CI	*P* value
Age category	⩽44 y	1.469	(1.00, 2.15)	.0484
	45-64 y	0.984	(0.68, 1.41)	.9285
	⩾65 y	Reference		
Gender	Male	0.827	(0.69, 0.99)	.0416
	Female	Reference		
Race/ethnicity	Hispanic	0.874	(0.62, 1.23)	.4401
	Black Non-Hispanic	0.784	(0.60, 1.03)	.0793
	Other Non-Hispanic	1.488	(1.08, 2.06)	.0165
	White Non-Hispanic	Reference		
Insurance type	Government	1.532	(0.68, 3.45)	.3038
	Medicaid	1.383	(1.06, 1.81)	.0176
	Medicare	0.808	(0.57, 1.15)	.2382
	Uninsured	1.353	(0.85, 2.16)	.2032
	Unknown/Missing	1.945	(0.75, 5.03)	.1699
	Commercial	Reference		
Number of beds	100 or less beds	0.413	(0.20, 0.85)	.0166
	500-999 beds	2.095	(1.72, 2.56)	<.0001
	1000 or more bed	3.584	(2.57, 5.00)	<.0001
	100-500 beds	Reference		
Rural-urban status	Counties in metro areas of fewer than 250 000 population	1.074	(0.76, 1.52)	.6907
	Counties in metro areas of 250 000 to 1 million population	0.859	(0.69, 1.07)	.1757
	Non-metro areas	0.778	(0.44, 1.39)	.3972
	Counties in metro areas of 1 million population or more	Reference		
Distance to hospital from home		0.999	(0.99, 1.00)	.6527
Opioids type	Heroin	1.049	(0.84, 1.31)	.6720
	Synthetics	1.422	(1.10, 1.85)	.0083
	Others	0.956	(0.71, 1.30)	.7742
	Natural/semi-synthetic opioids	Reference		
Alcohol	Yes	0.359	(0.13, 0.98)	.0453
	No	Reference		
Stimulant	Yes	0.799	(0.48, 1.33)	.3868
	No	Reference		
Year	2020-2021	0.846	(0.71, 1.01)	.0648
	2018-2019	Reference		

Table 3 includes the results from the multivariable logistic regression for leaving against medical advice. Younger age was highly significantly associated with higher odds (45-64 years: OR = 1.664, 95% CI: 1.25-2.22, *P* = .0005; ⩽44 years: OR = 2.519, 95% CI: 1.86-3.41, *P* < .0001) of leaving against medical advice compared to older age (⩾65 years). Male patients were much more likely to leave against medical advice (OR = 1.251, 95% CI: 1.09-1.44, *P* = .0014). Insurance status was also highly significant with patients on Medicaid (OR = 1.903, 95% CI: 1.56-2.33, *P* < .0001) and uninsured (OR = 2.129, 95% CI: 1.55-2.92, *P* < .0001) much more likely to leave against medical advice compared to patients on commercial insurance. Patients who had heroin overdose were more likely to leave (OR = 1.293, 95% CI: 1.05-1.59, *P* = .0148). Co-existing stimulant use was associated with higher odds of leaving against medical advice (OR = 1.499, 95% CI: 1.11-2.02, *P* = .0075). Years 2020 to 2021 was significantly associated with more leaving against medical advice compared to years 2018 to 2019 (OR = 1.265, 95% CI: 1.11, 1.44, *P* = .0003).

**Table 3.** Logistic regression for leaving against medical advice among opioid overdose hospitalizations.

Parameter		Odds ratios	95% CI	*P* value
Age category	⩽44 y	2.519	(1.86, 3.41)	<.0001
	45-64 y	1.664	(1.25, 2.22)	.0005
	⩾65 y	Reference		
Gender	Male	1.251	(1.09, 1.44)	.0014
	Female	Reference		
Race/Ethnicity	Hispanic	1.097	(0.85, 1.41)	.4674
	Black Non-Hispanic	1.134	(0.94, 1.36)	.1774
	Other Non-Hispanic	1.414	(1.09, 1.84)	.0102
	White Non-Hispanic	Reference		
Insurance type	Government	1.100	(0.54, 2.26)	.7957
	Medicaid	1.903	(1.56, 2.33)	<.0001
	Medicare	1.165	(0.90, 1.51)	.2482
	Uninsured	2.129	(1.55, 2.92)	<.0001
	Unknown/missing	3.493	(1.92, 6.34)	<.0001
	Commercial	Reference		
Number of beds	100 or less beds	0.649	(0.46, 0.91)	.0119
	500-999 beds	0.769	(0.66, 0.90)	.0008
	1000 or more bed	0.455	(0.29, 0.70)	.0004
	100-500 beds	Reference		
Rural-urban status	Counties in metro areas of fewer than 250 000 population	0.823	(0.64, 1.06)	.1309
	Counties in Metro areas of 250 000 to 1 million population	0.887	(0.76, 1.04)	.1354
	Non-metro areas	1.038	(0.76, 1.41)	.8123
	Counties in metro areas of 1 million population or more	Reference		
Distance to hospital from home		0.994	(0.99, 1.00)	.0325
Opioids type	Heroin	1.330	(1.14, 1.56)	.0004
	Synthetics	1.129	(0.92, 1.39)	.2528
	Others	1.029	(0.83, 1.27)	.7945
	Natural/semi-synthetic opioids	Reference		
Alcohol	Yes	0.696	(0.42, 1.17)	.1682
	No	Reference		
Stimulant	Yes	1.499	(1.11, 2.02)	.0075
	No	Reference		
Year	2020-2021	1.265	(1.11, 1.44)	.0003
	2018-2019	Reference		

## Discussion

This study examined the trends and outcomes of opioid overdose hospitalizations before and during the COVID-19 pandemic. Using the Pennsylvania Health Care Cost Containment Council (PHC4) hospital discharge database between 2018 and 2021, we found that there were no substantial changes in the number of overall opioid overdose cases and deaths at hospitals following the outbreak of COVID-19. However, we observed an increase in overdose cases involving synthetic opioids, which were associated with higher mortality rates. Moreover, we found that patients were more likely to leave against medical advice during the COVID-19 pandemic.

This study compared characteristics of patients with opioid-overdose-related hospitalizations in 2018 to 2019versus 2020 to 2021. We found no substantial increases in hospital opioid overdose cases. It may be associated with the reduced volume of hospital visits involving drug overdose during the peak of COVID.^2,26-28^ An analysis of Kentucky emergency medical services data indicated a prevailing hesitancy among patients with opioid overdose to visit hospital; there was a 71% increase in the number of opioid overdose ambulance runs in which patients refused to be transported to an emergency department.^2^ Another national study also described the declining trend in hospital admissions after the onset of the pandemic, plausibly due to cancellation and postponement of non-urgent, elective treatments.^28^ Many patients do not seek medical care at hospitals after overdose due to concerns about contracting and spreading the virus. Such fear of virus infection has been documented by an array of studies as one of the leading reasons why patients refuse to seek medical care at the hospital during COVID time,^26,29,30^ including patients with drug overdose. They might resort to other channels such as urgent care or die at home.^1^

Our finding that opioid overdose deaths at Pennsylvania hospitals in 2020 to 2021 were not significantly different from those in 2018 to 2019 adds to the literature with mixed evidence of the potential trend of opioid overdose mortalities between preceding and during COVID periods. Several studies observed no substantial increases in drug overdose deaths.^11,12^ An analysis of Massachusetts vital records and statistics suggested no heightened surges in overdose deaths between 2018 and 2020, although variability in overdose mortality trend by specific substance was noted before and during the COVID-19 pandemic.^11^ In contrast, a few studies suggested national and regional accelerations of drug overdose deaths during the COVID-19 pandemic compared to pre-COVID time, with an increase ranging from 30% to 49%.^1,31,32^ Further, a study of vital statistics data from 11 states in the US revealed great heterogeneity in opioid-related overdose deaths by state.^3^ Such geographic variations are potentially attributed to distinct data and populations investigated. They may also be driven by differential pre-existing patterns and trends of substance use prior to the pandemic and divergent states’ consequent responses to combat the drug overdose epidemic magnified by the COVID-19 pandemic.

Compared to studies that investigated vital statistics database to analyze overall overdose trends, our findings based on hospital discharge data highlighted that Pennsylvania hospitals did not experience surges in opioid overdose incidences and mortalities during the COVID-19. The prevalent fear of viral infection in hospitals might have deterred drug overdose patients from seeking hospital care, as indicated by a retrospective cohort study from Rhode Island. That study reported a decline in the proportion of drug overdose deaths in hospital inpatient settings from 12% in 2019 to merely 6% in 2020, along with an elevated rate of drug overdose deaths in personal residences from 13.2 per 100 000 person-years in 2019 to 19.7 in 2020.^1^ However, the absence of observed surges in opioid overdose cases and deaths in hospitals might be an alarming signal of a significant increase in overdose severity during the pandemic, which results in a higher likelihood of patients’ dying of drug overdose before being admitted to hospitals. This concern is substantiated by recent research demonstrating a continued acceleration of fentanyl-involved overdose deaths occurred outside hospitals following the outbreak of COVID.^33^

Our study found that the majority of patients admitted to hospital for opioid overdose were male, non-Hispanic White, and aged 45 to 64 years in both 2018 to 2019 and 2020 to 2021. Compared to the period of 2018 to 2019, the proportion of males in 2020 to 2021 significantly rose. A similar rise in overdose death risk among males during the pandemic was also documented by multiple regional analyses of drug overdose deaths.^1,34,35^ In contrast, a recent report of Massachusetts opioid overdose trends between 2018 and 2020 suggested a steady fraction of males dying from opioid overdose.^11^ It also revealed a slight increase in decedents’ average age,^11^ differing from our result of an unchanged age distribution of opioid overdose hospitalizations in Pennsylvania. In addition, we found younger age (⩽44 years) was associated with higher odds of opioid overdose mortality while male sex was estimated to have lower odds than their female counterparts. These results are partially consistent with a study of drug overdose mortalities before and during the COVID-19 using the National Vital Statistics System that suggested a positive correlation between monthly overdose mortality rate and younger age and male sex.^31^ Such heterogeneous findings may have resulted from differences in study period and population demographics investigated, given several deadly peaks of COVID-19 hit areas at different times and magnitudes.

Our study observed a marked decline in the proportion of non-Hispanic White patients in drug overdose hospitalizations, decreasing from 62.9% in 2018 to 2019 to 58.1% in 2020 to 2021. This trend warrants consideration within the context of the racial and ethnic composition of Pennsylvania’s population. As reported by the US Census Bureau,^36^ 74.5% of Pennsylvania’s population is non-Hispanic White. This indicates that, even prior to the COVID-19 pandemic, non-Hispanic Whites were significantly underrepresented in overdose admissions, an inequality that intensified during the pandemic. Simultaneously, there was a notable rise in the overrepresentation of racial and ethnic minorities in opioid use overdoses after the emergence of COVID-19, accompanied by higher odds of overdose death compared to their non-Hispanic White counterparts. Data from the CDC’s Wide-ranging Online Data for Epidemiologic Research (WONDER) database^37^ reveals that while the overall opioid overdose death rate per 100 000 in Pennsylvania escalated from 23.8 in 2018 to 32.8 in 2021, the increase was more pronounced among the non-Hispanic Black (from 24.5 to 51.7) and Hispanic (from 25.3 to 37.5) populations. Our findings are consistent with the majority of the literature, which underscores a shift in the substance overdose crisis from predominantly White to racial and ethnic minority populations—a trend that was evident prior to COVID-19 and intensified during the pandemic.^3,11,12,31,38^ A recent study of 2018 to 2021 national vital statistics suggested that Blacks, on average, experienced a 2.05% increase in monthly drug overdose mortality rate from 2018 to 2021, compared to only 0.96% for non-Hispanic Whites and that the pandemic even witnessed a particularly precipitous rise in Black overdose mortality rate (52%) versus in non-Hispanic White (45%).^31^ Reasons underlying the further heightened racial and ethnic inequities are multifaceted. Structural racism has long imposed barriers and challenges to racial and ethnic groups, including disinvestment in education, employment, and housing as well as limited access to health care services, substance treatment programs, and harm reduction interventions.^39^ Such longstanding historical socioeconomic marginalization experienced by racial and ethnic minority groups may render them more vulnerable to mental and behavioral disorders.^40-42^ These issues are frequently compounded by substance use and dependence, and are further aggravated by limited access to appropriate medications and treatments for substance abuse.^43^ Particularly, quarantine and social distancing during COVID-19 that were implemented to prevent the transmission of the virus probably further fuel their stress, anxiety, and depression,^8,44^ resulting in their heightened susceptibility to fatal overdoses.^45^ Also, stay-at-home orders may increase their probability of using substance alone and dying of overdose without any bystander as last resort.^1,2,46^ In addition, aspects of drug supply have also driven the shift of overdose mortalities from White to racial and ethnic minority populations. Historically, illicitly manufactured fentanyl has disproportionately affected Black communities as the primary driver of fatal overdose cases.^38^ The accelerated proliferation of fentanyl-adulterated or substituted heroin during COVID-19, particularly among urban Black and Latino communities, combined with the increased lethality of these illicit substances, may have further escalated the racial and ethnic inequities in the opioid crisis.^10,47^

During the pandemic, we observed an increase in the proportion of Medicaid enrollees presenting to hospitals due to drug overdoses, possibly due to economic instability during COVID and Medicaid expansion in Pennsylvania. Moreover, Medicaid coverage was associated with an elevated risk of overdose death, suggesting that people with financial stress, such as those experiencing job loss, may be more severely affected.^1^ People who are struggling financially may face greater barriers to accessing treatment and recovery support services for substance use disorders, and consequently, they are at an increased risk of death due to drug overdose. These findings underscore the urgent need for targeted policy interventions to alleviate the health inequities exacerbated by the pandemic.

Synthetic opioids were implicated in more overdoses during the COVID-19 pandemic and significantly associated with higher odds of opioid overdose death. This finding is in line with the nationwide escalating synthetic opioid crisis reported by a majority of previous studies.^1,3,10,32,38^ The CDC provisional data displayed a 53.1% increase in synthetic opioid overdoses from 2019 to 2020, as a key driver of the increasing overall opioid overdose mortalities during that period.^48^ The emergence of this synthetic opioid crisis is complex and attributable to an array of individual and structural factors on demand and supply side, including economic instability, mental health distress, health care inequity as well as increased lethality and variations of fentanyls and concomitant profit incentives, all of which have been magnified by the unanticipated lockdown owing to the COVID-19 pandemic. Curbing this rising tide of fentanyl-involved overdose deaths in the U.S. calls for more efforts at the federal and regional level. There is a large growing literature on strategies responding to this public health crisis.^49-51^

Our finding of lower odds of death with alcohol co-involvement is surprising, as prior research shows a higher fatality risk of co-involvement of alcohol and illicit substance. For example, DiGennaro et al described an increase in drug overdose deaths involving alcohol with fentanyl from 2018 to 2020 in Massachusetts.^11^ Several factors may account for this surprising result. Firstly, our study’s focus on hospitalized opioid overdose cases contrasts with research based on broader overdose incidence and mortality data from vital statistics. This difference in sample scope could contribute to the variance in findings. Indeed, the increasing severity of opioid overdoses suggests that more severe cases involving co-substances may result in mortalities before hospital admission. Consequently, the cases that do reach the hospital could represent less severe instances of co-involvement. Secondly, our methodology involved identifying substance involvement through the diagnosis by T code. The urgency often present in emergency settings could potentially lead to less comprehensive coding of alcohol co-involvement.^23^ Thirdly, our dataset does not provide information on the opioid dosages for patients with alcohol co-involvement. It is plausible that patients experiencing opioid overdose with alcohol co-involvement might have consumed lower opioid dosages compared to those without alcohol involvement.

Higher incidences of leaving against medical advice were observed during COVID time, which is consistent with the literature.^20^ A retrospective analysis of emergency department records in 3 U.S. hospitals documented a fall in the volume of ED visits and a substantial rise in walk-out rates during the early phase of the pandemic, including leaving against medical advice.^20^ A vast majority of studies found that patients’ leaving against medical advice was primarily attributed to their fear of contracting the virus at hospitals.^29,30,52^ Our results indicated that younger age, male sex, uninsured status or Medicaid coverage were positively associated with leaving against medical advice, which is in line with prior research for patients hospitalized with various diagnoses.^53-55^ Other risk factors for leaving against medical advice described in the literature included lower socioeconomic status,^56,57^ previous comorbidities (eg, HIV/AIDS and psychiatric illnesses),^58,59^ poor treatment experience at hospital,^60^ or lack of social support.^61^ Notably, leaving the hospital against medical advice can lead to serious health consequences, including inadequate or delayed treatments, increased risk of readmission, and higher mortality rates, which emphasizes the need for interventions to address this issue.

The findings of this study should be interpreted with several limitations in mind. Firstly, this was a single-state observational study, not a national evaluation. While the study offers valuable insights into the prevalence and outcomes of drug overdoses in hospital settings, contributing new evidence to our understanding of the evolution of the overdose epidemic during a major public health crisis, these findings may not be generalizable to national trends. This is due to substantial geographic variability in pre-COVID overdose trends, demographic shifts, healthcare systems, and state-level mitigation strategies. Second, we used hospital discharge database that was inevitably subject to inaccurate records, which may bias results. Third, information on the history of comorbidity, substance use disorder, or psychological distress for patients examined in this study was limited. Such pre-existing conditions may be strongly associated with the severity of their current drug overdose as well as treatment outcomes at hospitals. Fourth, the dataset lacked detailed information on the types and dosages of the drugs involved. Greater specificity regarding drug types and dosages could further illuminate the relationship between the opioid overdose and COVID-19 crises. Finally, the study used the most recent data available for the period ranging from 2018 to 2021. While this period offers an important opportunity to compare trends before and during the pandemic, it is necessary to continue investigating the long-term impact of COVID-19 on overdose epidemic as data beyond 2021 become available.

## Conclusions

This study based on Pennsylvania hospital discharge data from 2018 to 2021 revealed critical insights into the impact of the COVID-19 pandemic on opioid overdose hospitalizations. While the pandemic did not significantly alter the overall number or mortality rate of opioid overdoses in hospitals, it did prompt a noticeable change in patient behaviors, with a significant increase in the proportion of patients leaving against medical advice. Given the rise in opioid overdose death during the pandemic, it seems to imply that a larger number of patients succumbed to overdoses outside of hospital environments before they could receive medical attention due to higher overdose severity. Additionally, we observed a rise in the use of synthetic opioids during the pandemic, indicating possible shifts in substance use patterns in response to changing environmental factors. Further, the type of insurance coverage significantly influenced patient outcomes, with Medicaid insurance associated with higher odds of in-hospital death and leaving against medical advice.
